# Expanding research on the impact of financial hardship on emotional well-being: guidance of diverse stakeholders to the Emotional Well-Being and Economic Burden of Disease (EMOT-ECON) Research Network

**DOI:** 10.3389/fpsyg.2023.1196525

**Published:** 2023-07-28

**Authors:** Maria Pisu, Margaret I. Liang, Sarah D. Pressman, Carol D. Ryff, Minal R. Patel, Mustafa Hussein, Courtney P. Williams, Nora B. Henrikson, Yu-Mei Schoenberger, Laurel J. Pracht, Erin Bradshaw, Terrell Terri Carpenter, Amy Matthis, David L. Schwartz, Michelle Y. Martin

**Affiliations:** ^1^Division of Preventive Medicine and O’Neal Comprehensive Cancer Center, University of Alabama at Birmingham, Birmingham, AL, United States; ^2^Division of Gynecologic Oncology, Department of Obstetrics and Gynecology, Cedars-Sinai Medical Center, Los Angeles, CA, United States; ^3^Department of Psychological Science, University of California, Irvine, Irvine, CA, United States; ^4^Department of Psychology and Institute on Aging, University of Wisconsin-Madison, Madison, WI, United States; ^5^Department of Health Behavior and Health Education, School of Public Health, University of Michigan, Ann Arbor, MI, United States; ^6^Department of Health Policy and Management, Graduate School of Public Health, The City University of New York, New York, NY, United States; ^7^Kaiser Permanente, Washington Health Research Institute, Seattle, WA, United States; ^8^Ovarian Cancer Research Alliance, New York, NY, United States; ^9^Patient Advocate Foundation, Patient Insight Institute, Hampton, VA, United States; ^10^Carpenter Primary Healthcare, Memphis, TN, United States; ^11^American Diabetes Association, Alexandria, VA, United States; ^12^Departments of Radiation Oncology and Preventive Medicine, University of Tennessee Health Science Center, Memphis, TN, United States; ^13^Department of Preventive Medicine and Center for Innovation in Health Equity Research, University of Tennessee Health Science Center, Memphis, TN, United States

**Keywords:** emotional well-being, medical financial hardship, economic burden of disease, financial toxicity, network

## Abstract

The Emotional Well-Being and Economic Burden (EMOT-ECON) Research Network is one of six research networks funded by the National Institutes of Health (NIH) to advance research about emotional well-being (EWB), and the only one that focuses on addressing how economic burden due to disease or illness affects EWB. The network convened researchers, patients, patient advocates, health care providers and other stakeholders from across the US to discuss the significance of addressing the impact of the economic burden of disease on EWB, the complexity of this prevalent problem for patients and families, and the research gaps that still need to be studied to ultimately develop strategies to reduce the impact of economic burden of disease on EWB and health. Participants identified some important future areas of research as those investigating: (i) prevalent and relevant emotions for patients experiencing economic burden of disease and financial hardship, and how their broader outlook on life is impacted; (ii) constructs and contexts that influence whether the economic burden is stressful; (iii) strategies to deal and cope and their positive or negative effects on EWB and health; and (iv) multi-level and multi-stakeholder interventions to address economic factors (e.g., costs, ability to pay), administrative burdens, education and training, and especially patients’ emotional as well as financial status.

## 1. Introduction

Worry about affording medical care is highly prevalent among Americans (Weissman et al., 2020). About 60% of Americans stress about costs of health care and medications and/or medical bills including unexpected bills, and more than 50% worry they will not be able to pay for the health care services they may need in the future (American Psychological Association, 2018, 2019; Montero et al., 2022). It is also common to see media stories highlight exorbitant medical bills and medical debt that patients face. For example, a 2020 news article reported “on the verge of being intubated and put on a ventilator, the person [a COVID-19 patient] “gasped out” the question, “Who’s going to pay for it?” to their medical team” (Mahbubani, 2020). All medical conditions have economic consequences that result from the costs of medical care and treatment, other expenses indirectly related to care such as traveling to access doctors and care facilities, and potential loss of income (Pisu et al., 2018). The scientific literature over the past 10 years has defined and reported on medical “financial hardship” which refers to distress and difficulty in paying medical bills and accessing or using recommended medical care due to cost (Altice et al., 2017). Now, there is increased awareness of “financial toxicity” as a side effect of medical treatment equivalent to other physical toxicities such as nausea, pain, or fatigue (Zafar and Abernethy, 2013). In the United States, it is estimated that 137 million adults experience some form of medical financial hardship, including 28% of adults 65 and older and almost 47% of younger adults (Yabroff et al., 2019), across a spectrum from relatively manageable related distress to catastrophic expenses with medical debt being the number one cause of bankruptcy (Hamel et al., 2016).

Medical financial hardship is associated with worse mental and physical health, and leads to behaviors such as forgoing medical treatment and delaying health care that can be detrimental to health (Altice et al., 2017; Yabroff et al., 2019). However, its full impact on well-being has not been fully investigated yet. In particular, there is a great need to understand how medical financial hardship across its spectrum affects individuals emotionally due to notable and common increases in stress, depression, worry and other negative emotions that may result from such hardship (American Psychological Association, 2018, 2019; Barbaret et al., 2019; Chan et al., 2022; Montero et al., 2022). Similarly, there is a need to understand how these emotions and stress impact health outcomes (Pressman and Cohen, 2005; Diefenbach et al., 2008; Ryff and Singer, 2008; Irwin and Cole, 2011; Boehm and Kubzansky, 2012; Cohen et al., 2012; Pressman et al., 2019). Moreover, while there is growing research on the negative effects of financial hardship, much less is known about what to do to help with this issue. In cancer and other illnesses, patients manage the financial and emotional aspects associated with the economic burden of disease using strategies that can be problem- and/or emotion-focused (even while insured) (Head et al., 2018). It is necessary to understand the broader impacts these strategies have on patients and families. Similarly, interventions may be needed at community, health system, and policy levels to prevent medical financial hardship and its impact on well-being.

To advance knowledge in this area, the Emotional Well-Being and Economic Burden (EMOT-ECON) Research Network was funded to spearhead research and develop new insights about the impact of economic burden of disease on emotional well-being, with the ultimate goal of developing the strategies needed to reduce such impact. It is one of six research networks funded by National Institutes of Health (NIH) agencies to advance research about emotional well-being. Recently, emotional well-being has been defined as “a multi-dimensional composite that encompasses how positive an individual feels generally and about life overall, including both *experiential* features (emotional quality of momentary and everyday experiences) and *reflective* features (judgments about life satisfaction, sense of meaning, and ability to pursue goals that can include and extend beyond the self)” (Park et al., 2022). The economic burden of disease leading to medical financial hardship has the potential to impact both of these features. To guide its work, a Strategic Planning Meeting was convened virtually in October 2021, and an in-person Scientific Meeting was convened in January 2023. The goal of these meetings was to bring together diverse stakeholder groups from academic and other institutions across the US including researchers studying medical financial hardship and/or emotional well-being, patients and patient advocates, and health care providers, to guide ongoing and future work in these intersecting domains. This paper summarizes key discussions and recommendations from these meetings to inform the directions and priorities in the study of the economic burden of diseases and emotional well-being and future work of the EMOT-ECON network.

## 2. Meeting activities

### 2.1. Strategic Planning Meeting

**Figure 1:**
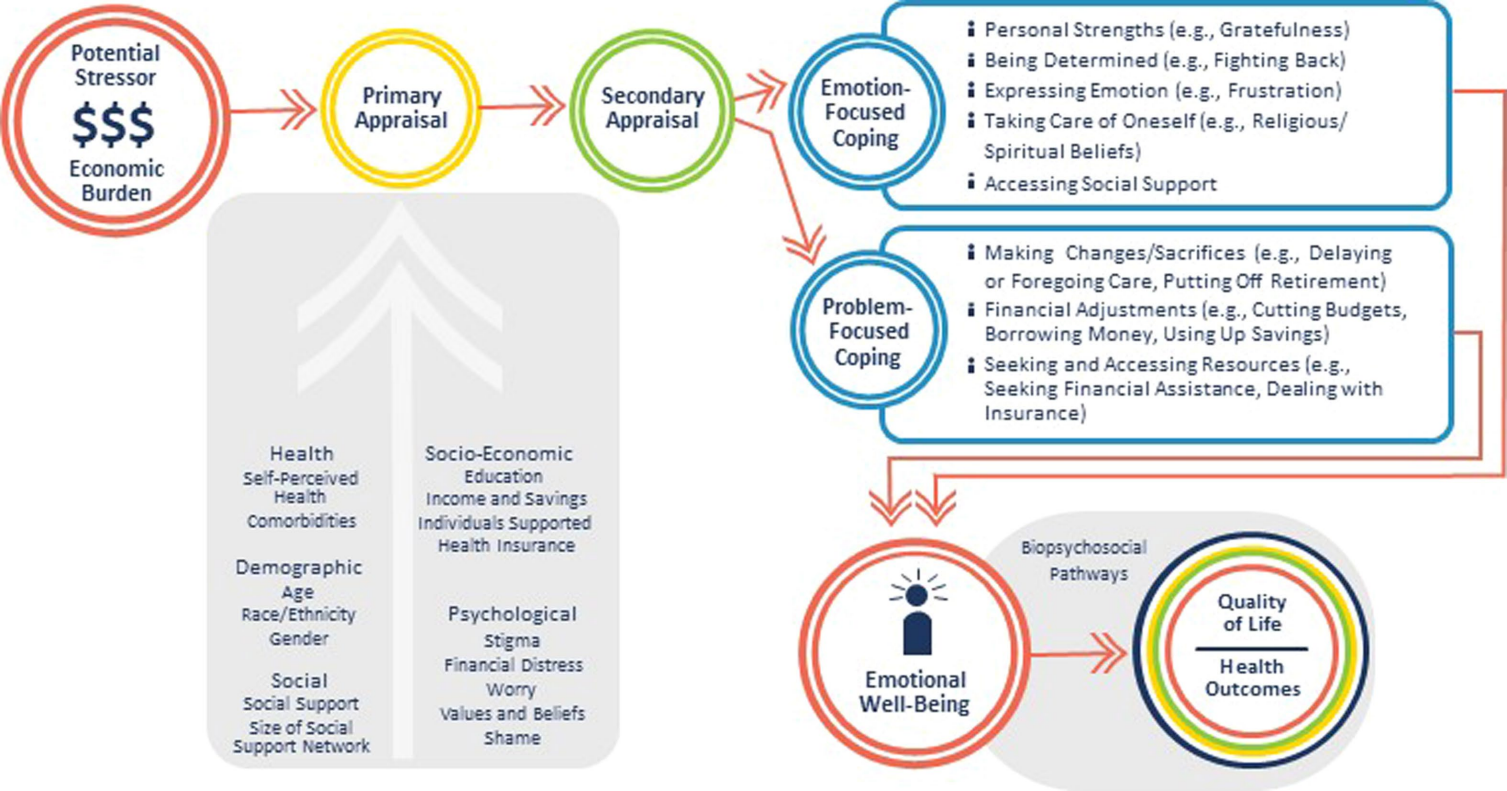
Conceptual framework showing the possible pathways in which the economic burden of disease impacts emotional well-being.

The four-hour virtual Strategic Planning Meeting included 13 invited participants from across the US including researchers, patient advocates, and health providers, plus EMOT-ECON investigators (MP, ML, MM) and staff. It started with a summary of existing literature and presentation of a conceptual framework to guide research of the network adapted from an established stress and coping theory (Lazarus and Folkman, 1984) (Figure 1). This framework acknowledges that the primary appraisal of the economic burden of disease as a stressor may be influenced by several health, demographic, social, economic, and psychological factors. Secondary appraisal may lead to different *strategies to deal or cope* with the stressor, selected based on the emotion-and problem-focused coping behaviors identified by Head et al. (2018) in a cancer population. Discussions on this framework were held along the three priority areas of EMOT-ECON, which include (i) Ontology and Measurement, i.e., identifying components of emotional well-being in the context of patients and families facing economic burden of disease; (ii) Mechanisms, i.e., the processes by which the economic burden of disease affects patients’ emotional well-being, and the processes by which emotional well-being affects health in this context; and (iii) Prevention and Intervention, i.e., potential interventions that may be relevant for minimizing the impact of economic burden of disease on emotional well-being. Discussions at this meeting were recorded and transcribed.

### 2.2. Scientific meeting

The in-person Scientific Meeting was a full day meeting with a total of 29 participants from academic and other institutions across the US including patients and patient advocates, health care providers, representatives from financial and media agencies, and researchers with expertise in psychology, sociology, economics, medicine, and health services research. The meeting included two sets of discussions with one moderator and 5–6 participants in each of 5 roundtables. Each set of discussions lasted about 40 min and the major themes from each roundtable were then shared with the larger group. Participants used sticky notes to write their answers to the posed discussion questions. These were collected by the moderators to guide the discussions and the summary reports to the larger group. All notes were subsequently transcribed. Summary reports were recorded.

For the first roundtable, participants were asked to discuss the different types of *strategies* used to deal or cope when facing different aspects of financial hardship, as well as their potential positive and negative effects on patients (direct effects), and caregivers, families, and others (indirect effects). For the second roundtable, attendees were asked to discuss *potential interventions* for primary, secondary and tertiary prevention. Primary prevention interventions were described as those that may be implemented to prevent the economic burden of disease from becoming a stressor. Secondary prevention interventions were described as those that may be implemented to identify and support patients who experience financial hardship to prevent an impact of financial hardship on emotional well-being. Finally, tertiary prevention interventions were described as those that may be implemented to manage poor emotional well-being resulting from economic burden of disease to avoid exacerbations and complications such as, for example, clinical depression.

## 3. Summary of Strategic Planning Meeting discussions

### 3.1. Ontology and measurement

Participants discussed limitations in the definition and measurement of both emotional well-being and financial hardship. For patients who experience medical financial hardship, unique and important elements that may not be appropriately assessed in existing measures of emotional well-being include the ability to get needed care and the ability to remain productive in day-to-day or work-related activities. With respect to economic burden, attendees proposed measuring a different construct. Acknowledging existing measures of overall financial well-being, they discussed measuring medical financial well-being rather than hardship, and then identifying protective factors that may preserve such financial well-being, which would benefit the field by shifting the focus on primary prevention. Similarly, participants noted that current measures of financial hardship relate to defined limited time points, e.g., over the past week or month, and miss the measurement of longer-term or chronic financial hardship, which may have different impacts on emotional well-being and health.

### 3.2. Mechanisms

Before discussing the mechanisms by which the economic burden of disease affects patients’ emotional well-being, participants discussed at length what influences whether the economic burden of disease becomes a stressor. First, participants discussed a hallmark of this stressor, the extreme *uncertainty and unpredictability*, compared to other financial stressors that may be more predictable, such as regular bills and expenses. The following characterize the economic burden of diseases:

- Unknown amounts of out-of-pocket expenses for medical care, even when patients are insured;
- Unknown timing of medical bills, including when patients are billed and when they should pay the bill;
- Unknown consequences of being unable to pay medical bills and worry about being unable to access needed treatment.

It was recognized that patients and their families may have varying baseline financial skills or self-efficacy to problem-solve when faced by these kinds of uncertainty.

Second, attendees identified the *difficulty of dealing with financial issues* as a specific and related stressor. Patients may have difficult and stressful interactions with insurance companies and health care billing offices, and may face the threat of having medical debt turned to collection agencies. These interactions add stress even at low levels of economic burden.

Participants then discussed how research should investigate the extent to which the economic burden of disease is perceived as a stressor across life circumstances. For example, this may depend on when the disease occurs during the lifespan, levels of available family or other support, cultural belief systems, socioeconomic status, or other social determinants of health. Given these varying circumstances, there may be a differential impact on the experiential and reflective components of emotional well-being described above (Park et al., 2022). Similarly, the extent to which economic burden of disease is perceived as a stressor may depend on disease prognosis and curability, which affect how patients prioritize health care in relation to the costs of care, and impact amount and duration of medical expenses and ability to work. Moreover, the extent to which economic burden of disease is perceived as a stressor may differ depending on aspects of the economic burden of disease, e.g., whether patients face high out-of-pocket expenses for care but no job loss vs. low or no high out-of-pocket costs of care but job loss.

With respect to mechanisms by which the economic burden of disease affects emotional well-being, participants discussed the role of what people do to deal or cope with the burden. First, upon reviewing the strategies listed in the conceptual framework, it was recognized that some of them have positive or negative effects on emotional well-being and health. For example, a problem-focused strategy cancer patients commonly adopt is to forgo medical care or to skip prescribed medications to reduce costs (Head et al., 2018). This may have negative consequences for disease progression and health, but it may also impact emotional well-being directly as patients may be acutely aware that this strategy is detrimental to their health. Second, attendees recognized that experiencing emotions such as anger or sadness may be appropriate responses to this burden, and thus contribute positively to emotional well-being. Third, participants discussed the importance of investigating differences in coping strategies and their positive and negative effects for patients with different socioeconomic status or living in different contexts, for example patients living in poverty or rural areas.

Lastly, participants discussed valuable areas of research to understand factors that could moderate the impact of economic burden of disease on emotional well-being. In particular, those discussed were: (i) comfort with uncertainty, (ii) literacy (health literacy, numeracy, insurance literacy, etc.) and the ability to understand costs and manage other personal economic challenges; (iii) resilience; (iv) personal empowerment in interacting with different medical and non-medical professionals to navigate financial hardship; (v) available resources such as support or insurance; and (vi) living context that may be characterized by existing policies or type of health care system, i.e., for example a system of universal health care coverage.

### 3.3. Prevention and intervention

Considering the stress caused by uncertainty, unpredictability, and dealing with financial issues, participants discussed potential benefits of interventions to improve knowledge of costs, such as financial and/or insurance education interventions. For example, financial counseling/navigation interventions are designed to link patients to needed resources, but could be extended to include efforts to inform patients about monetary costs or time off work, and even guide patients to choose care based on cost information, if appropriate. Entities providing such services could be within health care system or outside: for example, financial and/or insurance education programs may pair insurance representatives with patients to help them choose the “right” insurance, guide them through open enrollment processes, evaluate if supplemental insurance is needed, or simply help them understand what their insurance covers. Participants discussed ongoing projects where patients are paired with a financial counselor from a non-profit consumer education and training service group who help guide patients manage expenses while on treatment (Henrikson et al., 2022; Smith et al., 2022; Wheeler et al., 2022).

Participants considered whether individual-level interventions alone would be effective in reducing the impact of economic burden of disease on emotional well-being. Specifically, there was some discussion on whether stress reduction, coping-based interventions, or family interventions would be effective. Overall, it was acknowledged that financial hardship cannot be addressed solely at the individual level. Attendees considered what kind of studies could be done to inform healthcare policies to prevent economic burden of disease. For example, studies could compare the level of economic burden or financial hardship across states or other geographic areas with different drug pricing policies or health care or insurance market dominance.

### 3.4. Framework updates and recommendations

**Figure 2:**
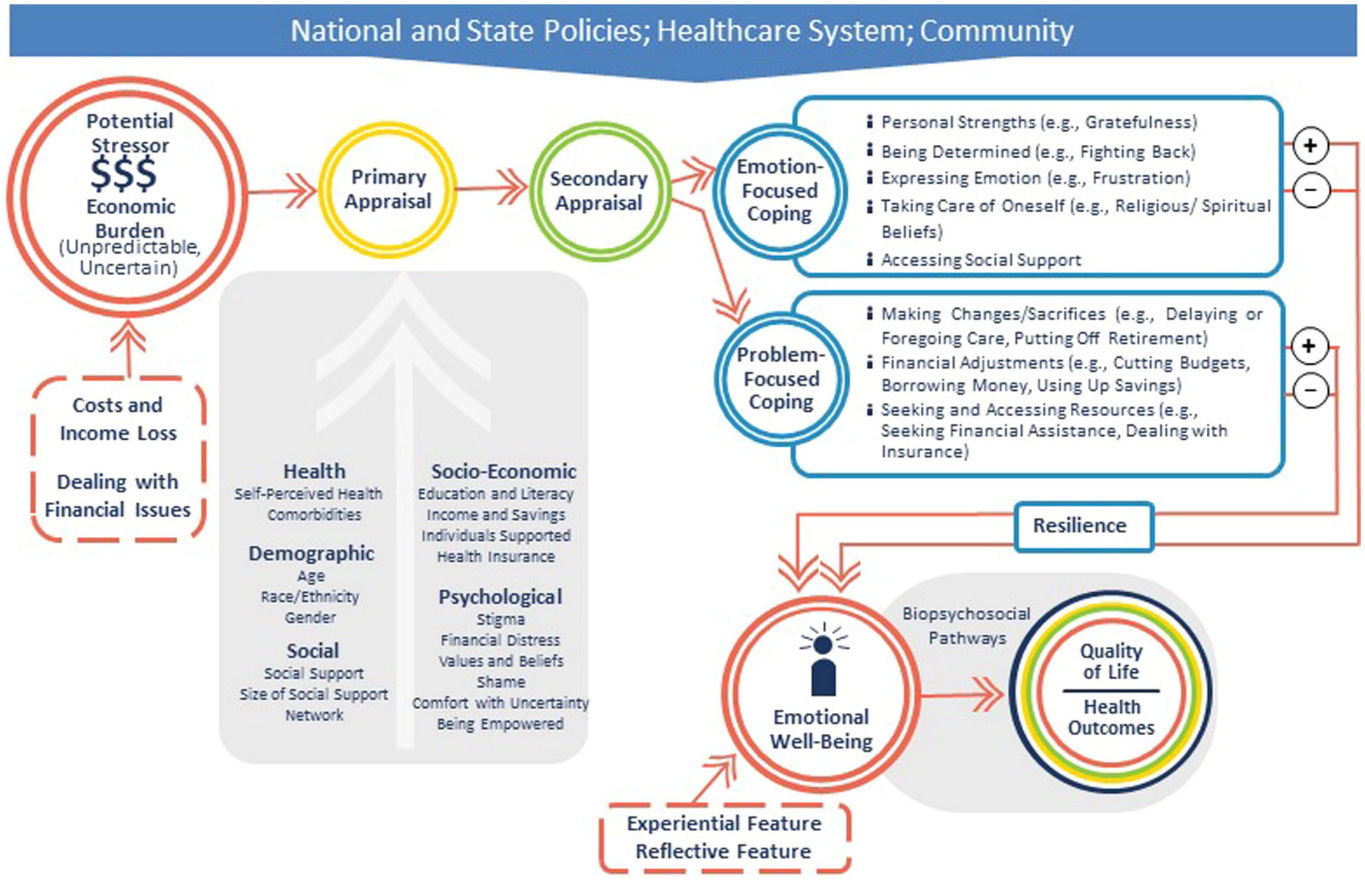
Updated conceptual framework after the EMOT-ECON Strategic Planning Meeting.

Based on the discussions, the network’s conceptual framework was updated as shown in Figure 2 to recognize the contexts in which financial hardship occurs and impacts emotional well-being, the characteristics and components of the stressor and emotional well-being, and the potential positive and negative effects of the “coping” strategies. Attendees recommended that the research of the EMOT-ECON network should grow organically without preset definitions of either emotional well-being or economic burden and that explorations of all aspects of the framework were needed and valuable. Overall, research on overwhelming costs of healthcare and other economic consequences of disease, and how they impact the emotional well-being of those subjected to such burden, will be important to bring public awareness to these problems.

## 4. Summary of scientific meeting discussions

### 4.1. Roundtable discussion on types of strategies to deal or cope with economic burden of disease, and potential positive and negative direct and indirect effects

Table 1 summarizes themes emerging from discussions about strategies which aligned with the emotion-focused and problem-focused coping illustrated in Figure 2. Attendees identified direct positive and negative effects that may be common across several types of strategies (e.g., reduced stress may be a direct positive effect of Managing Stress or Seeking Resources and Information, fatigue and energy expenditure could be direct negative effects of Self-advocating or Seeking Resources and Information) or unique to a strategy (e.g., specific positive and negative effects of Seeking Support). With respect to indirect effects, attendees proposed positive effects such as promoting preventive care in the family, or a transcendent effect by which the patient learns from the experience to help others. For negative indirect effects, attendees discussed the impact on family choices, for example on children’s college decisions, on caregivers’ work and health, and on relationship strains. Participants also highlighted the feeling of helplessness that not only patients and caregivers, but also others, in particular health care team members, may experience when observing someone deal with medical financial problems. All of these topics constitute important topics for future research on the economic burden of disease and its impact on emotional well-being.

**Table 1:** Some strategies to deal or cope with the economic burden of disease and potential effects identified in roundtable discussions at the Scientific Meeting of the EMOT-ECON network (Memphis, TN, January 2023).

Strategies	Examples	Effects	
		Positive	Negative
Emotional responses	Gratitude, Positive attitude, Manage emotions that prevent active coping, Ignore problem, Feel inadequate, Anger, Guilt, Fear, Shame, Constant grieving, Social disengagement	Discover own strength, Become a role model/example/source of hope	Decrease in emotional regulation, Reduced sense of self-worth, Overwhelming stress that makes it hard to take behavior action
Seeking support	Support from family friends, church, Prayer, Spending time with family, Tangible support with family finding extra job or loan	Become more socially connected, Increased sense of belonging/community, Renewed appreciation for the existing support, Increased sense of meaning, Winnow social networks to focus on people who are important	Worry about protecting family and friends from stress or from others’ judgments or negative reactions, Feel shame or stigma in asking for help, embarrassment about finances in addition to disease, the burden of making others feel more comfortable with the diagnosis, Manage expectations from others, Compare self to others (social media), Relationship erosion
Self-advocating	Self-advocating when dealing with administrative burden	Increased sense of control, Increased resilience, Sharpened self-efficacy skills, Increased awareness of own resourcefulness	Fatigue/feeling overwhelmed from having to self-advocate
Managing stress	Manage stress through meditation, exercise, art therapy, humor and other stress relieving activities, Taking personal time, Substance use and abuse	Reduce stress/hormones (biological benefits), Positive lifestyle changes	Substance use dependency
Seeking resources and information	Seek/utilize financial assistance programs, information about anticipated costs and to navigate complexities of insurance, Track medical and other costs, Get treatments from other countries, Seek cheaper treatments, Find “donors” or use crowdfunding platforms	Decrease debt and burden, Increased sense of control and ability of maintaining normalcy, Reduce stress/hormones, biological benefits	Fatigue, Extra energy expended, Stress of making the case for worthiness to donors, Donor fatigue based on erroneous belief that costs stop after treatment
Making economic adjustments	Borrow money from friends/relatives/financial institutions, Seek payment plans and discounts, Deplete savings, Sacrifice leisure activities, Find extra jobs, Apply for disability	Gaining general financial skills, Obtain extra money, Reduced medical costs, Ability to pay medical and other bills	Increased debt, Guilt of spending family money, Disempowerment and lack of control, Feel inadequate (cannot provide for family), Credit rating and long-term impact lasting for years and impacting family members
Making medical care adjustments	Forgo or delay care, Go off treatment without consultation, Ration or stop medicine doses	Reduced treatment costs	Deterioration of health, Suboptimal care, Worry about effects on health
Changing life perspective/goals	Find other positive things in life, Change work/life goals	Become a role model/example/source of hope, Re-assess values and priorities	Not able to have life milestones; Not able to play usual role in family or social circle

### 4.2. Roundtable discussion on potential primary, secondary, and tertiary prevention interventions

Participants discussed interventions at society or community, policy, and health care system levels across the three prevention approaches (i.e., primary, secondary, tertiary). Table 2 summarizes interventions for primary and secondary prevention. Discussions recognized that interventions need to reduce health care costs, increase the ability to pay, reduce administrative burden, and raise awareness, education, and training about financial hardship for patients, caregivers, and the workforce of health care, financial and other institutions. Attendees also recognized that these efforts need to go hand in hand with interventions to strengthen mental health, tailoring them to meet patients where they are mentally and financially, so as to provide the best chance of successfully reducing the impact of economic burden. Attendees supported screening for financial hardship, but also for anxiety and depression, recognizing that patients with mental health problems may be less able to deal with practical and financial problems. They also supported financial navigation and coaching in health care and other systems, as well as strengthening peer support to address economic burden.

**Table 2:** Some potential prevention interventions identified in roundtable discussions at the Scientific Meeting of the EMOT-ECON network (Memphis, TN, January 2023).

Levels	*Primary Prevention Interventions* To prevent the economic burden of disease from becoming a stressor	*Secondary Prevention Interventions* To prevent an impact of financial hardship on emotional well-being
Society/Community	Early education through schools or media awareness campaigns on: - Costs of care and financial toxicity - Financial education and financial literacy - Health insurance - Emotion-based coping Employment of financial coaches in banks, insurance companies, and other institutions, with training in costs of care and medical debt	Education on insurance literacy and costs of care for patients and caregivers Personal financial coaches in hospitals/clinics Establishment of programs in financial institutions to train employees to help people with severe disease manage overall debt (medical and not).
Policy	*Insurance*: Universal healthcare; Revisiting health insurance benefit/policy to design coverage that minimizes patient burden; Health insurance reform including elimination of premium increases during illness, establishing disease specific out-of-pocket maximum, covering certain conditions fully, offering Medicaid supplements after reaching out-of-pocket maximum regardless of personal resources; Close coverage gap for Social Security Disability Insurance and Medicare. *Income-related*: Guaranteed income during severe illness and for clinical trial participation; Employment protections; Automatic eligibility for disability for specific diagnoses without burdensome eligibility process and automatic renewals. *Pharmaceutical companies*: Policies to lower costs of drugs; Drug price policy reform. *Research*: Funding for research to gain evidence for policy change.	*Insurance*: Ensure coverage for mental health.
Health care system	*System-level changes*: *Costs*: Reduced operation costs; Provision of transportation and more local services to decrease travel costs; Implementation of institutional simplification to reduce administrative burden. *Care*: Standardized provision of information on support systems and expected cost estimates early in treatment; *Mental health*: Establishment of screening for depression and anxiety as patients with mental health problems may be less able to deal with practical and financial problems *Workforce*: Establishment of financial navigation/counseling; Increased providers’ awareness of economic burden of disease and of resources available to help patients; Creation of medical school curricula to raise awareness about costs of care *Patients*: Patient education on asking questions about resources, Removal of stigma	*System-level changes*: Establishment of universal screening for financial issues and risk of job loss; Systematic queries of social needs and screening patients based on available metrics at the institutions, i.e., prior use of financial assistance or payment plans, debt with institution, high utilization of ED; Establishment of a stratification system for people who need more or less intervention support; Establishment of workflow to deal with crisis, i.e., for patients with severe financial distress. Optimization of follow-ups and referral to available resources post financial hardship screening; Reducing administrative burden, and wait times while improving referral systems; Consideration of billing pauses and billing forgiveness tied to payment; Lower patient costs/fees; *Care*: Provision of consolidated care to reduce costs and address all patients’ needs; Provision of treatment plans that include costs; Interventions based on patient’s profile and/or previous history of mental and financial stress; Ensuring patients are comfortable accessing provider care team (doctors, navigators, etc). *Workforce*: Provision of training on cost conversations; Establishment and training of financial navigators or coaches. *Patients*: Establishment of support groups with patients with financial hardship experience or training existing support groups to talk about costs of care and financial hardship; Education on disability benefits; Informational support to stop avoidance behaviors like ignoring payments due; Financial and insurance literacy training; Education for caregivers.

Discussions for tertiary prevention to manage poor emotional well-being resulting from economic burden of disease and prevent exacerbations and complications reflected on the effects of economic burden and the strategies to deal and cope with this burden reported in Table 1. Participants proposed strengthening mental health workforce and support with interventions to address clinical depression, to target positive affect and also to promote a more holistic, eudaimonic well-being that incorporates meaning and purpose (Ryff, 2017). Moreover, participants discussed the need to support caregivers facing burnout and helplessness.

## 5. Conclusion

Researchers, patients, health care providers, patient advocates, and other stakeholders, bring unique perspectives to the task of understanding the impact the economic burden of disease on emotional well-being and ultimately on health. This cross-disciplinary lens made for unusually energizing and creative discussions at the Strategic Planning and Scientific meetings of the EMOT-ECON network, attesting to the unique value and power of this approach. Overall, although not an exhaustive list, participants identified some important future areas of research, which included: (i) aspects of emotional well-being relevant to patients experiencing economic burden of disease and financial hardship, both in terms of what are prevalent and relevant emotions, and how the broader outlook on life is impacted; (ii) constructs and contexts that may influence, or protect from, perceiving the economic burden as stressful; (iii) ways in which patients deal or cope with the medical financial hardship across different contexts and populations with positive or negative effects on emotional well-being and health; and (iv) interventions at multiple levels and from multiple stakeholders that address economic factors (e.g., costs, ability to pay), administrative burdens, education and training, and especially patient’s emotional as well as financial status.

Discussions at these EMOT-ECON meetings and the updated framework align with current research on financial hardship. For example, recent research has started to recognize the complexity of such hardship which is not only due to high costs of care or reduced ability to work and earn an income, but to uncertainty and the difficulty of dealing with financial issues. Cancer patients in Gharzai et al. (2021) described the impact of having limited knowledge of the treatment course, of the costs and work limitations to be incurred, and the financial adjustments patients make through treatment. Lyman and Kuderer (2020) discuss the “abuse” and “torture” associated with dealing with the health care system when patients are not able to meet financial responsibilities, especially for the most vulnerable patients. In a German population with a different health care system from the US, Lueckmann et al. (2022) found that bureaucracy had a significant impact on whether patients experience financial distress, with patients feeling helpless due to time-consuming and complex processes, incomprehensible decisions by authorities and agencies, and lack of knowledge about rules and regulations when dealing with these entities. Thus, as emerging from the EMOT-ECON meetings, future areas of research may include the investigation of personal characteristics like comfort with uncertainty, empowerment in dealing with financial issues, or literacy (e.g., health literacy, health insurance literacy, numeracy), and their role in how patients deal with the characteristics and components of the economic burden of disease, how they cope, and what the effects of that coping are on emotional well-being. The need to maintain focus on structural problems (cost of healthcare, insurance bureaucracies) is also key going forward.

The strategies identified in our meetings to deal and cope with the economic burden of disease build on those identified in previous literature (Head et al., 2018; Banegas et al., 2019; Kayser et al., 2021). Several frameworks recognize the potential negative impact of creative but medically non-advisable problem-focused strategies like lower adherence to treatments, delayed or forgone care (Altice et al., 2017; Carrera et al., 2018; Lentz et al., 2019). However, the literature has not clearly delineated the pathways by which financial hardship, directly and through these strategies, affects emotional well-being, health, and quality of life. Some studies have examined the mediator effects of social support and limiting care due to costs on the relationship between financial toxicity and quality of life (Hastert et al., 2019; Hallgren et al., 2020). Santacroce and Kneipp (2019) explicitly includes the biological response to stress in the pathway from financial hardship to quality of life outcomes of parents of pediatric oncology patients, which is critical because this increased stress can change disease course and related outcomes (Cohen et al., 2007). The themes reported in Table 1, although not exhaustively, provide an initial roadmap to guide future research on understanding the occurrence and extent of proposed direct and indirect effects of strategies to deal with the economic burden of disease. Importantly, our discussions highlighted the impact these strategies may have in the present, but also on the long-term financial status and outlook on life and future well-being of patients and families. Further research on all these aspects and on mechanisms by which the economic burden of disease impacts emotional well-being, and then overall health, is required to evaluate the full impact of financial hardship due to disease. Previous literature on emotions and stress has shown how these may have important implications for health outcomes by significantly altering physiological processes, for example, impacting the immune system, increasing inflammation and susceptibility to infection or by leading to poor health behaviors, such as poor sleep quality, unhealthy eating habits, and reduced physical activity (Diefenbach et al., 2008; Irwin and Cole, 2011; Cohen et al., 2012). Positive emotions, however, can be protective by leading to improvements in health behaviors, undoing some of the health harms of stress and together with other types of psychological well-being (e.g., purpose, good relationships) leading to healthier physiological profiles (e.g., healthier blood pressure and immune function), better longevity and disease morbidity, and in some cases, higher disease survival (Pressman and Cohen, 2005; Ryff and Singer, 2008; Boehm and Kubzansky, 2012; Pressman et al., 2019). Thus, it is important to understand how emotions and stress deriving from the economic burden of disease impact physiology and health behaviors, and if intervening on these leads to better health outcomes in patients experiencing medical financial hardship.

Participants highlighted how interventions to prevent or mitigate financial hardship are not limited to providing financial support either by reducing costs or providing financial navigators or counseling. Some of these interventions have been or are currently being tested (Patel et al., 2021; Smith et al., 2022), and will be important tools to prevent the impact of economic burden of disease on emotional well-being. Discussions at the EMOT-ECON meeting highlighted the concurrent need for mental health interventions to prevent financial hardship in patients who may be too stressed to deal with financial issues, or to mitigate its impact for those already experiencing it. In fact, several strategies were proposed to systematically identify and intervene on individuals based on both their emotional and financial status. The effectiveness of this approach across a spectrum of financial hardship severity would be an important area of future research. Moreover, as previously advocated (Yabroff et al., 2020), participants recognized that, to make meaningful changes, addressing medical financial hardship requires a multilevel approach and multiple stakeholders’ commitment starting with raising awareness and educating individuals before they become patients.

In summary, discussions at the EMOT-ECON network meetings highlighted the significance of addressing the impact of the economic burden of disease on emotional well-being and beyond, the complexity of this prevalent problem for patients and families, and the research gaps that still need to be studied. The EMOT-ECON network will support researchers who tackle these research gaps to advance understanding of both the economic burden of disease and emotional well-being, and help build the knowledge base to ultimately develop strategies to reduce the impact of economic burden of disease on the emotional well-being, and ultimately the health, of patients and their loved ones.

## Author contributions

MPi and MM developed the concept and obtained funding for the EMOT-ECON network. MPi wrote the manuscript. MM and ML contributed critical revisions to the first draft of the manuscript. All authors contributed to organization, conduct of the EMOT-ECON meetings, manuscript revisions, read, and approved the submitted version.

## Funding

This study was funded by the National Center for Complementary and Integrative Health (NCCIH), the Office of Behavior and Social Sciences Research (OBSSR), the Office of Disease Prevention and National Institutes of Health Office of the Director (U24AT011310).

## Conflict of interest

The authors declare that the research was conducted in the absence of any commercial or financial relationships that could be construed as a potential conflict of interest.

## Publisher’s note

All claims expressed in this article are solely those of the authors and do not necessarily represent those of their affiliated organizations, or those of the publisher, the editors and the reviewers. Any product that may be evaluated in this article, or claim that may be made by its manufacturer, is not guaranteed or endorsed by the publisher.
